# MicroRNA-155 Implication in M1 Polarization and the Impact in Inflammatory Diseases

**DOI:** 10.3389/fimmu.2020.00625

**Published:** 2020-04-15

**Authors:** Sergiu Pasca, Ancuta Jurj, Bobe Petrushev, Ciprian Tomuleasa, Daniela Matei

**Affiliations:** ^1^Department of Hematology, Iuliu Hatieganu University of Medicine and Pharmacy, Cluj-Napoca, Romania; ^2^Research Center for Functional Genomics, Biomedicine and Translational Medicine, Iuliu Hatieganu University of Medicine and Pharmacy, Cluj-Napoca, Romania; ^3^“Octavian Fodor” Institute of Gastroenterology and Hepatology, Cluj-Napoca, Romania; ^4^The Oncology Institute Prof. Dr. Ion Chiricuta, Cluj-Napoca, Romania; ^5^Department of Gastroenterology, Iuliu Hatieganu University of Medicine and Pharmacy, Cluj-Napoca, Romania

**Keywords:** macrophage, polarization, microRNA-155, inflammation, M1

## Abstract

Macrophages are known to have an impact in cytokine signaling in the myriad of organs in which they reside and are classically known to be either pro-inflammatory (M1), anti-inflammatory (M2). Different classes of signaling molecules influence these states, of which, microRNAs represent key modulators. These are short RNA species approximately 21 to 23 nucleotides long that generally act by binding to the 3′ untranslated region of mRNAs, regulating their translation, and, thus, the quantity of protein they encode. From these species, microRNA-155 was observed to be of great importance for M1 polarization. Because of it’s major implication in M1 polarization microRNA-155 was shown to be implicated in different inflammatory diseases. To name a few, microRNA-155 was shown to be modified in patients with asthma and to correlate with asthma symptoms in mouse model; it has been shown to modulate the activity of foam cells and influence the dimensions of the atherosclerotic plaque and it has also been shown to be of crucial influence in transducing the signal of LPS in septic shock. Because of this, the current review aims to offer an overview of the role of microRNA-155 in M1 polarization, the implication that this poses for the pathophysiology of inflammatory diseases and the potential therapeutic possibilities that this knowledge might bring. Currently, microRNA-155 has been used in clinical trials as a marker of inflammation, but the question remains if it’s inhibition will be useful in inflammatory diseases, as other products might have a better cost/benefit ratio.

## Introduction

Macrophages have been known for their implication in the immune system, either through their phagocytic and/or antigen presenting abilities. Nonetheless, another important property of this type of cells is represented by their role in immune signaling. At the basis of their inflammatory functions stays the concept of macrophage polarization, which, in a simplified manner, is considered to be classic/proinflammatory (M1) or alternative/anti-inflammatory (M2) (1). The transition between different states of polarization is regulated by several classes of molecules, of which microRNAs present high importance. These are short RNA species of approximately 21 to 23 nucleotides that bind to different RNA species modifying their quantity or changing the rate at which they are translated. Other mechanisms through which microRNAs can act as regulatory molecules are represented by DNA binding at the promoter regions, influencing transcription or direct protein binding (2).

Because of the high number of microRNAs and targets that these species have, we decided to further focus on the implication of microRNA-155 in inflammatory diseases.

## Macrophage Polarization

The continuum of macrophage polarization starts with the M0 polarized macrophage. One protein with key importance in macrophage polarization is represented by *PU.1*, which opens the chromatin conformation of macrophage-specific genes and further allow transcription factors to act in a cell-specific manner (3–6).

**FIGURE 1:**
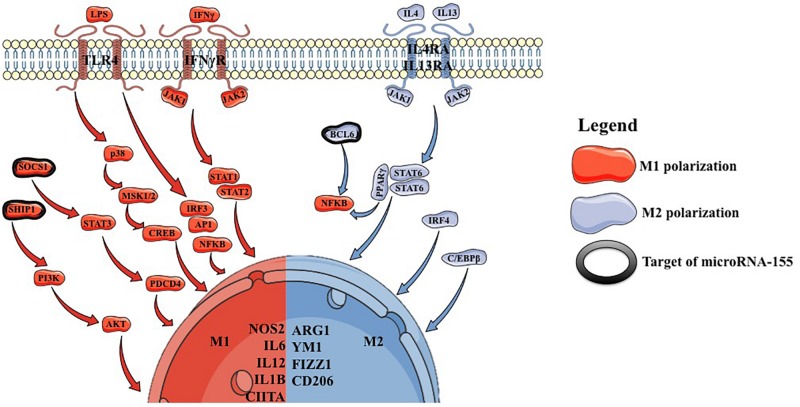
Signaling pathways implicated in M1 and M2 polarization.

Conventionally, it was considered that macrophages can polarize either to an M1 or an M2 phenotype (Figure 1). Nowadays it is known that macrophages take a dynamic state between these two phenotypes.

M1 polarization can be induced *in vitro* by stimulating M0 macrophages with *IFNγ* (interferon γ) or with lipopolysaccharide (LPS), which mimics *in vivo* conditions of M0 to M1 transition. *IFNγ* acts on the *IFNγR*, which further recruits *JAK1/2* leading to the formation *STAT1/STAT2* heterodimers, which act as a transcription factor for *NOS2*, *MHC2* and *IL12* (7, 8). LPS acts through *TLR4* (toll like receptor 4), stimulating *IFNα/β* autocrine signaling and leading to the activation of *STAT1* and *STAT2*, with the formation of *STAT1/STAT2* heterodimers, which act as a transcription factor for *NOS2*, *CIITA* and *IL12*, leading to a similar phenotype as generated through *IFNγ* direct stimulation. Additionally, *TLR4* can also lead to the activation of NFKB and mitogen associated protein kinase (MAPK) pathways with similar effects (9). These stimulatory factors lead to the deposition of M1-specific transcription factors, followed by the upregulation of *IL12* and downregulation of *IL10* (10, 11).

M2 polarization is induced by a combination of *IL4* and *IL13* stimulation of M0 macrophages (12). This leads to *STAT6* homodimer formation followed by the upregulation of *MRC1*, *CD206*, *FIZZ1* and *YM1* (13, 14). Additionally, *STAT6* represents a cofactor for *PPARγ* leading to an inhibition of NFKB pathway (15–18). *C/EBP* was shown to increase the transcription of *ARG1*, *IL10* and *MRC1* (19–21). Moreover, mice lacking *C/EBP*, were shown to have a reduced number of M2 macrophages, but with no influence on M1 macrophages (22).

As M1 and M2 macrophages have opposite effects it has also been shown that *STAT1* has opposite effects compared to *STAT6* regarding the transcription profile they induce (23). Interestingly, *JMJD3* was shown to play roles in both M1 and M2 polarization. In the case of M1 polarization, *JMJD3* was shown to respond to LPS and induce *IL12* and *CCL5* (24). In the case of M2 polarization, it was shown that the macrophages of mice lacking *JMJD3* are unable to undergo M2 polarization (14).

Although the general classification tends to picture these two macrophage polarization phenotypes as steady states, *in vivo* these represent a continuum and can transit from one to another. In this direction, one study has shown that after a period of LPS stimulation, the M1 response genes require a more intense stimulus for the same activation, while the response to *IL10* stimulation is kept (25). Another mechanism through which the proinflammatory processes are controlled is represented by a multistep process activated by the NFKB pathway, which leads to the upregulation of microRNA-155 and an initial upregulation loop through microRNA-155 mediated inhibition of *SHIP1*, with subsequent activation of the PI3K/AKT pathway. In parallel the NFKB pathway upregulates microRNA-146a, which inhibits *IRAK1* and *TRAF6*, leading to the resolution of the inflammatory process (26).

## MicroRNA-155 Biogenesis and Mechanism of Action

MicroRNA-155 is located on chromosome 21 and is encoded by the gene *MIRHA155*. Its biogenesis is similar to the classical microRNA maturation. Firstly, it is transcribed in the form of pri-microRNA, which undergoes 3′ guanine capping and poly adenylation, after which it is processed in pre-microRNA by DROSHA. The pre-microRNA form is exported from the nucleus through *XPO5* using a GTP-dependent process. In the cytoplasm, the pre-microRNA is processed by DICER, which cleaves the hairpin, resulting in the formation of a double-stranded RNA, which is loaded in *AGO2*. This, in turn, interacts with other proteins forming the RISC complex. In this complex the passenger strand is cleaved and only the guide strand remains loaded (2, 27, 28). Regarding microRNA-155, it is generally considered that the 5p form is the guide strand, thus being the one kept in the RISC complex after processing. Nonetheless, there have been studies showing that the 3p form can also act as a guide (29).

## The Regulation of MicroRNA-155 Expression and Its Effect on Target Genes

MicroRNA-155 regulation is highly linked to its role as an immune modulator. This is being shown by its rapid increase in macrophages in infection or other inflammatory processes. These processes are characterized by the stimulation of *DAMP*, *PAMP*, *IL1A*, *IL1B*, *TNFα*, and *IFNγ*, while being suppressed by antiinflammatory molecules, e.g., *IL10*, *TGFβ*, and glucocorticoids (30–33). Therapeutically, it has been shown that glucocorticoids can indirectly inhibit microRNA-155 expression by inhibiting NFKB pathway (34, 35). MicroRNA-155 implication in inflammatory processes can also be inferred from the regulatory sites the *MIRHA155* gene presents, these being influenced by NFKB pathway, TGFβ pathway through *SMAD4*, and through *IL10* and *IFNγ* stimulation (33, 36).

It has been shown that microRNA-155 is upregulated by the stimulation of TLR4 and action of *IFNγ*, known drivers of M1 polarization. MicroRNA-155 role in promoting inflammation can also be observed as it inhibits *INPP5D*, an inhibitor of PI3K/AKT pathway, which is needed to relay the signal from the TLR4 signaling. Another important target of microRNA-155 affects is represented by *SOCS1*, which inhibits type 1 cytokine receptor/STAT pathways. Moreover, it has been shown that microRNA-155 inhibits *BCL6*, an inhibitor of NFKB pathway, with important implications in the signaling of foam cells (37–39).

Primary macrophages derived from mice lacking microRNA-155 and the RAW264.7 cell line with an induced deficit in microRNA-155 have been shown to present a resistance to LPS stimulation, associated with an increase in *INPP5D* expression (32, 37, 40). Not only does microRNA-155 stimulate M1 polarization, but it has also been shown to inhibit M2 polarization through the inhibition of *IL13* and *IL4* pathway components, like *IL13RA* and *C/EBP* (41–45).

## MicroRNA-155-5P Vs MicroRNA-155-3P, Is There Only One Guide?

Generally, it is considered that microRNA-155-5p represents the guide strand as it targets a larger array of transcripts and it is considered to be the form with a higher thermodynamic stability. Nonetheless, there have been studies showing that microRNA-155-3p can also play biologically important roles (46, 47). Although the evidence is not as vast as in the case of microRNA-155-5p, there have been studies showing that microRNA-155-3p also has a role in promoting inflammation. For example, in the case of plasmacytoid dendritic cells, the initial response to *TLR7* stimulation has been shown to be represented by microRNA-155-3p upregulation and subsequent upregulation of IFNα/β autocrine signaling. These processes add a parallel to LPS stimulation of macrophages with potential similar signaling effects (48, 49).

## MicroRNA-155 in Inflammatory Diseases

**FIGURE 2:**
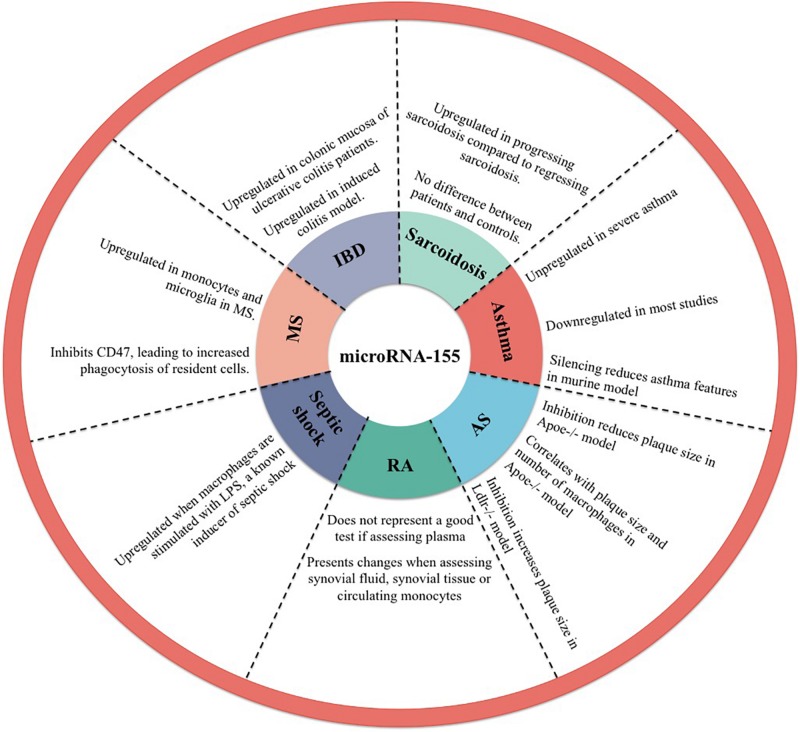
Implication of microRNA-155 in inflammatory diseases. AS, atherosclerosis; RA, rheumatoid arthritis; MS, multiple sclerosis; IBD, inflammatory bowel disease.

MicroRNA-155 is implicated in the M1 polarization in a variety of inflammatory diseases with the following representing some examples in this direction (Figure 2).

### Asthma

Classically, asthma is characterized by a chronic imbalance between type 2 and type 1 immune responses and chronic hyper-reactivity of the airways. In this condition a myriad of microRNAs have been identified with implication in either M1 or M2 polarization (50, 51). Human airway smooth muscle cells from asthmatic patients were shown to present elevated levels of microRNA-155 compared to controls (52). Conversely, some studies have shown that microRNA-155 presents lower levels in breath condensates, plasma, nasal mucosa, epithelial cells and sputum of asthmatic patients (50, 52–55). Others have shown that severe asthma patients present elevated plasma microRNA-155 when compared to mild-moderate asthma patients and to healthy controls. Interestingly, in this study there was no difference observed between the microRNA-155 plasma levels of mild-moderate asthma patients compared to healthy controls (56). Moreover, it has been shown that microRNA-155 silencing in an ovalbumin asthma mouse model reduces asthma features, this, in turn, showing a potential therapeutic path for the future (57).

### Atherosclerosis

Atherosclerosis is characterized by the formation of atheromatous plaques on arterial walls with subsequent reduction in blood flow, hypertension and an increased risk of embolus formation. One key cell with importance in the pathophysiology of atherosclerotic plaques is represented by the macrophage, which accumulate lipids and forms foam cells with a proinflammatory influence and leading to the evolution of the atherosclerotic plaque (58). Studies on *APOE*^–/–^ mouse models with partial carotid ligation showed a decreased plaque formation and lower number of infiltrating macrophages when the microRNA-155 levels were inhibited (37). These results were reproduced by others showing that downregulating microRNA-155 in *APOE*^–/–^ mouse model lowers the dimensions of the atherosclerotic plaque (59). The effect of microRNA-155 on atherosclerotic plaque formation has been explained through the modulation of the SOCS1-STAT3-PDCD4 pathway (60, 61). Considering these results, it could be though that microRNA-155 silencing might have a role in the future as therapy for decreasing the size of atherosclerotic plaques or to slow their evolution. In contrast to the above mentioned results, *LDLR*^–/–^ mice models showed that transplantation with microRNA-155 deficient macrophages leads to an increase in the size of atherosclerotic plaque and leads to a decrease in *IL10* levels secreted by peritoneal macrophages (62).

### Rheumatoid Arthritis

Rheumatoid arthritis (RA) is characterized by progressive joint inflammation leading to joint damage and subsequent disability. One important hint that macrophage present an important role in this condition is represented by an upregulation in *IL6* that these patients present, as this represents an important cytokine secreted by M1 macrophages (63). In RA patients it has been observed that microRNA-155 is upregulated in synovial tissue and synovial fibroblasts (64). Murata et al. did not observe any difference between the peripheral blood concentration of microRNA-155 of rheumatoid arthritis patients and healthy controls (65). Nonetheless, others have shown that microRNA-155 is upregulated in peripheral blood mononuclear cells (PBMCs) from patients with RA (66, 67). These results show that it is more likely that microRNA-155 concentration in the synovial fluid or in the cells involved in the process of joint inflammation is an indicator of disease, rather than the plasma levels of microRNA-155.

### Septic Shock

Septic shock represents the effect of LPS stimulation of the *TLR4* receptor on macrophages with a subsequent discharge of proinflammatory molecules leading to severe hypotension and organ dysfunction. Because it is known that LPS stimulation of macrophages lead to M1 polarization, it is highly probable that microRNA-155 also plays a role in this septic shock (68). *In vitro*, microRNA-155 was observed to be upregulated in THP1 cells when stimulated with LPS (69). In a BALB/c mouse model it has been observed that LPS stimulation increases microRNA-155 expression in the liver. Moreover, this increase in microRNA-155 was observed to be inhibited by pretreatment with dexamethasone, known as a therapeutic approach in septic shock and an indirect inhibitor of microRNA-155 (70).

### Multiple Sclerosis

Multiple sclerosis is characterized by the progressive demyelination of axons with subsequent reduction in the functions deserved by them. One important mechanism through which demyelination occurs is through myelin phagocytosis by the local microglia (71). In multiple sclerosis patients it has been shown that microRNA-155 is upregulated in CD14+ monocytes and in microglia of multiple sclerosis patients compared to healthy controls (72). In a multiple sclerosis mouse model it has been observed that the focal lesions contain microRNA-155 upregulation. This, in turn, has been shown to inhibit the expression of CD47 in brain resident cells, leading to an increase in myelin phagocytosis by macrophages (73).

### Inflammatory Bowel Disease

Inflammatory bowel diseases are characterized by an inflammatory state of the mucosa and submucosa in the case of ulcerative colitis and of all the layers of the bowel in the case of Crohn’s disease with subsequent functional impairment of these organs. Currently, there is not a precisely known pathogenesis, but inflammation was shown to play an important role and immunosuppressive and antiinflammatory therapies were shown to have an effect on these diseases (74). In a murine model of induced colitis it was shown that microRNA-155 is upregulated (75). Moreover, microRNA-155 was observed to be upregulated in the colonic mucosa of patients with ulcerative colitis (76).

### Sarcoidosis

Sarcoidosis is a disease characterized by a systemic granulomatous response of unknown origin. An important microscopic clue to the pathophysiology of the disease is represented by the presence of giant cells, formed by the fusion of multiple macrophages, at the center of the granulomas. This, in turn, shows the possible inflammatory state caused by these cells (77). MicroRNA-155 was observed to be upregulated in progressing sarcoidosis compared to regressing sarcoidosis, but no difference was observed when comparing sarcoidosis patients with healthy controls (78).

## Clinical Trials

At the time of this review, 26 clinical trials were found when searching on ClinicalTrials.gov for the term miR155. The search term microRNA-155 yielded fewer results, which were overlapping with the previous search term. Of those, 20 clinical trials include patients with inflammatory diseases and mention using microRNA-155 as a marker for assessing the activity in these diseases. Of those, one trial assessed microRNA-155 in asthma (NCT02719145); none assessed microRNA-155 in patients with atherosclerosis, but there were three trials assessing it for cardiovascular diseases (NCT02605512, NCT02997462, and NCT04277390); two trials assessed microRNA-155 in RA, one as a response to tocilizumab, an anti *IL6* antibody (NCT03149796) and one as a response to tofacitinib, a JAK inhibitor (NCT03815578); one trial assessed microRNA-155 dynamics in days 1, 2, 5 and 7 after septic shock onset (NCT02464371); one trial plans to assess the levels of microRNA-155 in the serum of patients with multiple sclerosis (NCT04300543) and no trials plan to evaluate the levels of microRNA-155 in patients with inflammatory bowel disease or sarcoidosis.

## Conclusion

MicroRNA-155 represents both a target of proinflammatory signals and an initiator of inflammation with an important impact in M1 polarization. Moreover, it has been repeatedly shown that microRNA-155 upregulation represents an important signal in various inflammatory diseases, with some clinical trials harboring this association as a biomarker for inflammation. As there are no clinical trials using microRNA-155 inhibition as an intervention in inflammatory diseases it can be asked if microRNA-155 will ever be used as more than a biomarker. MicroRNA-155 might have some interventional clinical trials in the future for inflammatory disease, but with the reserve that it might not have a suitable cost/benefit ratio or that other already existing compounds might have a more important effect in modulating inflammation.

## Author Contributions

SP, AJ, and BP wrote the manuscript. CT and DM coordinated the team members and made appropriate changes to the final version of the manuscript. All authors agreed on the final version of the manuscript.

## Conflict of Interest

The authors declare that the research was conducted in the absence of any commercial or financial relationships that could be construed as a potential conflict of interest.
